# Prevention of Osteoarthritis Progression by Statins, Targeting Metabolic and Inflammatory Aspects: A Review

**DOI:** 10.31138/mjr.32.3.227

**Published:** 2021-09-30

**Authors:** Behzad Heidari, Mansour Babaei, Behnaz Yosefghahri

**Affiliations:** 1 Mobility Impairment Research Centre, Babol University of Medical Sciences, Babol, Iran; 2 Department of Medicine, Division of Rheumatology, Rouhani Hospital, Babol University of Medical Sciences, Babol, Iran

**Keywords:** osteoarthritis, statin therapy, symptom, incidence, progression

## Abstract

**Background and purpose:**

Several traditional risk factors of atherosclerosis such as age, obesity, and altered lipid metabolism are shared with osteoarthritis (OA). Metabolic abnormalities and atheromatous vascular disease are linked with systemic inflammation and progression of OA. Hence, treatment of OA with statins is expected to improve metabolic abnormalities and prevent OA progression. Many studies which have addressed this issue found inconsistent results. This review aims to elucidate the effect of statins in OA by summarizing the existing data.

**Methods:**

Potential studies in English language published in Medline/PubMed, Scopus and Google Scholar since 2000 were searched by using keywords such as osteoarthritis, statins, progression, treatment, prevalence, synovitis, pain. Fourteen papers were found to be relevant and were summarised.

**Results:**

Data regarding symptomatic effect of statins in OA are scarce and the results varied from no effect to a small improvement or even increased risk of pain in knee OA. However, most studies on the incidence and progression of OA found a significant decreased risk of incident OA, as well as reduced risk of radiographic progression in statin users vs. non-users. Factors such as patient adherence, duration of treatment, and higher cumulative statin doses were associated with greater efficacy.

**Conclusion:**

Existing data indicate a preventing effect of statin therapy on OA progression. However, unless a formal meta-analysis with weight analysis is made, a conclusion cannot be drawn.

## INTRODUCTION

Osteoarthritis (OA) is known as an age-related chronic progressive disease and a leading cause of disability as well as impaired physical activity in the elderly population. At present, OA is considered as an inflammatory disease in which both aging and injury-induced joint damage are contributing to the development of synovial inflammation and cartilage degeneration.^1^ Several conditions including mechanical, metabolic and inflammatory factors are involving in the aetiology of OA. The pathophysiological association between metabolic abnormalities in particular obesity and OA is not limited to the loading effect of increased body mass index, but also with their link with biochemical/biological mechanisms, which also explain the association between obesity and OA of non-weight-bearing joints, such as hand OA.^2,3^ Thereby, clinical improvement of KOA after weight reduction is not solely to the consequence of mechanical load reduction but also to metabolic changes and decreased levels of inflammatory biomarkers. Consequently, in subjects undergoing bariatric weight loss surgery, pain relief which appears during the first months after surgery, still continues several months thereafter even in the absence of weight loss maintenance.^4^

The inflammatory process in OA originates from adipose tissues in particular from local fat pads and is linked to proinflammatory metabolic factors.^5^ Fat tissue as an endocrine organ secrets a group of pro-inflammatory mediators^6,7^ including cytokines (IL-1, IL-6, IL-8, TNF-a) and adipokines (leptin, adiponectin, resistin, visfatin). These mediators are released from the local and/or systemic adipose tissues due to damages of joint tissues consequent to trauma and/or overuse.^3^ Obesity and metabolic syndrome (MetS) as prominent risk factors of OA are highly prevalent in the general population, and supply greater sources of adipokines from adipose tissues and thus provide higher propensity for the development of OA.^3,8^ Pathophysiology, clinical features and progression of structural changes in OA are linked to release of mediators from adipose tissues.^9^ Irrespective of inflammation, vascular pathology also plays an important role in the initiation and progression of OA.^10^

## OSTEOARTHRITIS AND VASCULAR DISEASES

OA is shared with several traditional risk factors of atherosclerosis, such as age, obesity, altered lipid metabolism, and hypercholesterolaemia. Deposition of lipid in the joint tissues has been observed at the early stage of OA prior to histologic changes. Similar to atherosclerosis, hypercholesterolaemia can cause oxidation and deposition of lipids in tissues and lead to cartilage damages.^11,12^ It has been shown that chondrocytes aging is closely related with progression of cartilage degeneration, and the results of experimental studies indicate that statins protect chondrocyte aging and articular degeneration.^13^ In obese people who are at a greater risk of OA, adipose-tissue derived mediators such as TNF-α, IL-6 and IL-1 are produced at higher levels. These cytokines induce and regulate inflammatory immune responses in cartilages through inhibition on the synthesis of proteoglycans and type II collagen. Furthermore, adipokines promote endothelial cell dysfunction, adhesion of monocytes, vascular remodelling, and foam cell formation in the arterial wall and thus contribute to obesity-associated vascular complications.^14^

Epidemiological studies suggest a link between atheromatous vascular disease and progression of OA. In population of the Rotterdam study, the plasma levels of atherosclerotic markers in women with knee osteoarthritis (KOA) were higher than those without KOA, suggesting an association between atherosclerosis and KOA.^15^ Also, in participants of the Chingford study, there was an inverse association between HDL cholesterol and radiographic hand OA.^16^

Vascular pathology impairs blood flow and result in subchondral bone ischemia, osteocyte death and bone resorption with subsequent cartilage degradation.^11,17^ In postmenopausal women, there is a relationship between atherosclerosis and OA: in particular, hand OA and knee OA. This issue indicates that atherosclerosis and OA are shared at least in a number of risk factors or pathophysiological processes.^18,19^ In one study comprised population with advanced KOA, patients with coexistent diabetes and hypertension had greater subchondral bone loss as compared with those without these conditions suggesting a possible association between OA and metabolic abnormalities.^20^

However, the association between vascular disease and OA is complex, because the consequences of vascular pathology on OA per se cannot be differentiated from the contribution of individuals risk factors of vascular disease such as diabetes, obesity, hypertension, lipids or from the consequences of metabolic syndrome on OA.

## OSTEOARTHRITIS AND METABOLIC ABNORMALITIES

Several metabolic abnormalities such as obesity, diabetes, insulin resistance, and dyslipidaemia are related with OA, and these conditions are also associated with increased risk of atherosclerotic disease.^12,21^ This issue indicates that OA is not solely an age- or weight-related disease, but it is really a metabolic syndrome-associated disease with a low systemic inflammation which originates from metabolic abnormalities. The presence of both inflammation and abnormal metabolic condition leads to cartilage degeneration and disease progression.^22–24^ An association between dyslipidaemia and elevated risk of knee pain and clinical KOA in middle-aged or older adults has been also observed in a case-control study by Zhou et al.^25^ In this study knee pain was 1.36 to1.43 times, and clinical KOA was 1.49 to 1.57 times more prevalent in subjects with hyperlipidaemia or those using lipid lowering drugs as compared with subjects who had normal lipids. Similarly, a population-based case-control study found 1.37 (1.28–1.47) times greater risk of hyperlipidaemia in patients with hand OA.^26^ Two meta-analyses have also revealed a positive association between OA and hyperlipidaemia.^27^ In animal model of OA, there was an association between high fat diet and cartilage degradation independent of body weight.^28^ These observations suggest a possible contributive role of metabolic syndrome and lipid abnormalities in the initiation and development of OA. Therefore, statins with both cholesterol lowering and anti-inflammatory properties expected to provide a beneficial effect against OA progression.

## EFFICACY OF STATINS IN OSTEOARTHRITIS

Currently, treatment of osteoarthritis is focused on relieving pain and controlling the associated factors of disease progression and exacerbations. In subjects with irreversible damages, joint replacement with prosthesis is recommended.^2^

OA is a multifactorial disease with heterogeneous phenotypes in terms of clinical manifestations and aetiologies. Classification of OA to subgroups according to structural, etiological and epidemiological phenotypes will help to identify a specific subgroup of patients who display inflammatory characteristics. This subgroup is expected to gain more benefit from anti-inflammatory treatment.^29^ Therefore, patients who have synovitis (inflammatory phenotype) are anticipated to attain greater benefit from anti-inflammatory drugs which suppress adipose tissue-derived mediators of inflammation.^6^ So far, the results of treatment with anti-inflammatory drugs in particular disease-modifying anti-rheumatic drugs in OA have not been promising. Even at early stage of OA, treatment with potent anti-inflammatory treatment by using systemic and intra-articular biologic agents to inhibit TNFα and IL-1β, were disappointing.^22^ However, data in this context are scarce and limited to a few studies.^30–33^ Only in one RCT of 60 patients with erosive hand OA, treatment with adalimumab for 12 months provided a small but significantly less radiological evolution of erosive changes without a clear clinical benefit.^30^

Given a contributive role of dyslipidaemia as well as inflammation in the pathogenesis of both atherosclerosis and OA, using statins (HMG-CoA reductase inhibitors) in the treatment of OA seems to be sounds and logical. Statins exert an anti-inflammatory effect via inhibiting the synthesis of non-sterol isoprenoid compounds. Furthermore, statins improve endothelial cell function, stabilize atherosclerotic plaques, increase nitric oxide bioavailability, exert antioxidant properties and reduce smooth muscle cell proliferation. These properties are independent of their lipid-modifying effects.^34^ In addition, statin medications reduce production of IL-6 and IL-1β and provide an immunomodulatory effect through T cell activation as well as inhibition of IFNγ-inducible class II major histocompatibility complex (MHC) expression.^35^ A meta-analysis of 15 studies revealed that treatment of rheumatoid arthritis patients with atorvastatin and simvastatin reduced significantly both serum lipids levels and markers of inflammation.^36^ Since statins are commonly recommended for primary and secondary prevention of coronary heart disease, they can be also utilised for the prevention of cartilage degeneration and OA progression by both their anti-inflammatory and lipid lowering properties.^37–39^ In particular, statins exert an additional influence on the release of cytokines, chemokines and MMP from chondrocytes, synoviocytes and infiltrating immune cells, and thus might play a role in the regulation of joint anabolism and catabolism.^40^

For these reasons, the potential of statins in the prevention of OA progression has been examined in many longitudinal studies by comparison of statin users and non-user participants, but the results were inconsistent across different studies because of variations in study population, design of studies, and outcome measures.

To elucidate the effect of statin on OA and to summarise the existing data regarding preventive effect of statins in OA, English language databases were searched to identify potential studies which have been published in Medline/PubMed, Scopus, and Google Scholar since 2000 by using keywords such as osteoarthritis, statins, progression, treatment, prevalence, synovitis, pain, relief. In addition, the references of the selected papers and review studies were also searched to find eligible papers. All relevant sources were critically analysed to ensure diversity in the sources and to avoid bias.^41^ A total of 125 studies were found, in which 55 papers were irrelevant based on the titles and were thus excluded. Among the 70 remaining studies, 41 were not eligible and removed. A total of 29 full texts which have been identified for further review, out of which only 14 papers were eligible for analysis. The selected articles were categorized according to study designs and the results were presented under subheadings in relation to effects of statins on joint symptoms and function, development, incidence, and progression of OA.

## INFLUENCE OF STATINS ON SYMPTOMS OF OA

The results of several studies which have compared OA symptoms between statin users and non-users vary from no effect to a small improvement of statin therapy on OA symptoms.^42–44^

A few studies have found an increased risk of pain or poor function of the knee joint in statin users (**Table 1**). Efficacy of statins on OA symptoms is expected to be mediated through suppression of synovitis. However, synovitis is not the only cause of pain in KOA; obesity, muscle weakness, vitamin D deficiency, and mechanical factors also contribute to the development of pain in KOA which are not responsive to statins.^45–47^ In addition, muscle pain and weakness are adverse effects of statins therapy which have been reported in high percentage of statin users. These complications can mimic OA symptoms and confound the results.^48^

**Table 1.** Studies on the association between statins use and symptoms of osteoarthritis (OA).

**Author(year) ^References^**	**Study design, population**	**Aims of study**	**Results**
Veronese (2019)^42^	A longitudinal study, a 4-year follow-up of 4,448 community-dwelling adults from the Osteoarthritis Initiative Study.	To determine whether statin use is associated with lower risk of radiographic OA radiographic symptomatic KOA ^a^ and pain	Using statins was not associated with lower risk of pain worsening, incident OA, or symptomatic KOA. However, statin use > 5 years and using atorvastatin were associated with lower risk of developing pain by RR = 0.91(0.83–0.997) and rosuvastatin was associated with higher risk of developing pain by RR = 1.18 (1.12–1.24).
Peeters et al. (2015)^43^	Data from middle -aged and older female participants of the Australian Longitudinal Study on Women’s Health.	To determine the association between statin use and joint symptoms.	In middle-aged women statin was weakly associated with poor physical function by OR = 1.29(1.07–1.55) and poor self-reported health by OR = 1.35 (1.13–1.61). There was no association between statin use with joint pain/stiffness.
Riddle et al. (2013) ^44^	Data provided for 2207 participants of the Osteoarthritis Initiative Study with confirmed or suspected KOA. Statin users accounted for 6.7% of the sample in year 1 and 16.4% in year 4.	To assess the effect of statin on pain, function and structural changes in knee joint.	Statin use was not associated with improvements in knee pain, function or structural progression after a 4 - year follow-up period.

a KOA = Knee osteoarthritis

Nonetheless, in community-dwelling adult participants of the Osteoarthritis Initiative study, statin treatment for more than 5 years as well as use of atorvastatin alone was associated with a lower risk of developing pain, whereas use of rosuvastatin was associated with increased risk of developing pain.^42^

To summarise, data regarding the association between statin therapy and symptoms of osteoarthritis are limited. Only one study found a decreased risk of pain development in atorvastatin users or taking any statins for more than 5 years.

## ASSOCIATION OF STATIN USE AND INCIDENT OA

Seven observational studies have compared incident OA between statin users and non-users (**Table 2**). Two earlier studies found lower risk of developing clinical OA in statin users vs. nonusers.^49,50^ In a study by Kadam et al.^50^ development of OA within 2 years of statin therapy was 18% lower than non-users, and in subjects taking statins for more than 4 years, incident of clinical OA in statin users was 40% lower than non-users. A recent 7-year longitudinal study found a dose-dependent lower incidence of spinal degenerative joint disease (DJD) in statin users as compared with non-users.^51^ Conversely, in a retrospective cohort study, the risk of incident OA in statin users was higher. However, the results of latter study should be considered with limitation, because, incident of OA was not the primary objective of this study, and persons who received statins even for a short period of 3 months were also included. Short-term exposure to statins is not only expected to affect the rate of incident OA, but could also be a source of overestimation.^52^

**Table 2.** Studies on the association between using statins and incident osteoarthritis (OA).

**Author(year) ^References^**	**Study design, population**	**Aims of study**	**Results**
Wang et al. (2020) ^55^	Meta-analysis of 11 observational studies on the association between statin and OA comprised 679,807 participants.	To determine the association of statin use with the incidence and progression of OA.	There was no significant association between statin use and symptomatic or radiologic OA as well as with incidence and progression of OA. However, subgroup analysis showed opposite effects of atorvastatin and rosuvastatin on OA.
Burkard et al. (2018)^54^	A propensity score-matched cohort study of 233,608 statin initiators vs. the same number of non-initiators from the participants of the UK-based Clinical Practice Research Data line.	To determine the association between initiation of statin prescription and hand OA.	Over a maximum follow-up duration of 5.5 years, there was no different in the incident hand OA between statin initiator (patients initiated with ≥1 statin prescription) and non-initiators.
Cheng et al. (2018)^51^	Longitudinal study of 7238 statin users and 164 454 non-users were followed for 7 years	Association between statin dosage and development of spinal degenerative disease (DJD).	In hypercholesterolemia patients who took higher cumulative dosage of statin (11900–28000 mg) compared with a group taking < 5400 mg), development of spinal DJD reduced significantly by HR = 0.83 (0.70–0.99). At dosages of > 28000 mg spinal DJD reduced further by HR= 0.81(0.68–0.97).
Valdes et al. (2014)^53^	A case-control study, comprised 661 statin users and 2510 non-user participants of the GOAL study.	To determine the association of statin use with generalized nodal OA.	After adjustment for confounders, statin usage was not associated with nodal OA, hip OA or knee OA, but was associated with lower rate of GOA phenotype.
Kadam et al. (2013)^50^	Cohort design with a 10-year follow-up period comprised 16,609 cardiovascular patients.	To determine the association between statin use and occurrence of clinical OA by comparison of statin users and non-users.	Use of statin was associated with significant reduction in clinical OA. Larger cumulative dosage was associated with greater reduction (18% in parsons using statins within 2 years and 40% in persons taking statins for > 4 years vs. non-users.
Mansi et al. (2013)^52^	A retrospective cohort study comprised 12,980 statin users and 45,997 non-users taking statins for more than 3 months vs. non-users.	To determine the incidence of various musculoskeletal and neoplastic diseases in statin users and nonusers.	Over a 4-year follow-up period, using statin for at least 3 moths was associated with higher rates of OA by OR =1.26 (1.19–1.33), and arthropathies by OR = 1.20 (1.12–1.27) as compared with non-users.
Chodick et al. (2010)^49^	A population -based cohort study comprised 211,627 and 193,770 statin users (individuals who began statin therapy between 1998 and 2007).	To determine development of incident RA and risk of OA among adults with persistent and nonpersistent statin users	Over 9-year follow-up, in highly persistent patients who were covered with statin for at least 80% of the follow-up period, incidence of RA decreased by HR = 0.58 (0.52–0.65) and risk of OA decreased by HR= 0.85 (0.81–0.88) as compared with nonadherent patients.

Alternatively, two observational studies found no association between statin use and OA.^53,54^ Similarly, a meta-analysis of 11 observational studies found no significant association between statin use and incident OA. However, in subgroup analysis, taking atorvastatin was associated with a decreased risk of incident OA, whereas, using rosuvastatin was associated with increased risk of OA.^55^

To summarise, the results of studies on the association between use of statins and incident OA varied from reduced risk of OA to no effect or increased risk of incident OA. Factors such as adherence to statin use, duration of treatment with statin and cumulative dose of statins were associated with lower rate of incidence OA.

## ASSOCIATION BETWEEN STATINS USE AND PROGRESSION OF OA

Efficacy of statins in the prevention of OA progression has been shown in several studies (**Table 3**). In participants of the Rotterdam Study, over a follow-up duration of 6.5 years, statin use was associated with more than 50% decreased risk of progression of knee OA by OR = 0.43 (0.25–0.77), but there was no effect on progression of hip OA.^56^ A few limitations of this study include absence of knee OA at baseline in most participants, loss of a significant number of patients during follow-up period, and availability of final radiographs only for the 38% of the included participants. These issues could be sources of underestimation of OA progression. Furthermore, in this study, assessment of radiographic progression was confirmed by Kellgren and Lawrence score (K-L), which is not sensitive enough. Over 6 years of follow-up period only 3% of statin users and 7.3% of non-users showed structural changes.

**Table 3.** Studies on the association between statin use and progression of osteoarthritis (OA).

**Author(year) ^References^**	**Study design, population**	**Aims of study**	**Results**
Cook et al. (2020)^58^	Data from a large population-based clinical database comprised 151,305 participants, who underwent a THA/TKA^a^ surgery from 1988 to 2016.	To determine the association between time of initiation and duration of statins exposure with risk of revision of hip/knee arthroplasty after total hip/knee arthroplasty (THA/TKA).	Exposure to statins within 1 year and 1–5 years following THA/TKA reduced risk of revision arthroplasty by HR = 0.82 (0.75–0.90). Exposure > 5 years vs. < 1 year was associated with further risk reduction by HR = 0.74 (0.62–0.88).
Haj-Mirzaian et al. (2019)^57^	A longitudinal PSM ^b^ study, retrospective analysis of prospectively collected data from Osteoarthritis Initiative cohort	To assess the incidence of radiographic KOA and progression of joint space narrowing (JSN) in stain users vs. nonuser over 8 years of follow-up with regard to Heberden nodes (HN)	Only in HN - positive patients statin use was associated with decreased risk of JSN by 46%, but not in HN negative
Eymard et al. (2018)^61^	Longitudinal follow-up of 336 participants from placebo arm of the SEKOIA trial. Statin users accounted for 21.1% of participants.	To assess the impact of statin use on incidence and radiologic progression of knee osteoarthritis in patients with symptomatic disease.	Taking statin was associated with increased risk of JSN ≥ 0.5 mm over 3 years independent of confounding factors.
Michaëlsson et al. (2017)^62^	Longitudinal study by pooled analysis of 4 population-based large cohorts comprised 132,607 persons.	Association between statin use and risk of progression of knee osteoarthritis (KOA) by comparison of users vs. non-users	Risk of consultation or surgery of KOA or hip OA did not differ between non-users and current users of statins. Over a 7.5-year follow-up, the risk did not change by dose and duration of treatment. Furthermore, risk of KOA in parsons taking statins for less than 1 year did not differ with those > 3 years
Riddle et al. (2013)^44^	Data provided for 2207 participants of the Osteoarthritis Initiative Study with confirmed or suspected knee OA. Statin users accounted for 6.7% of the sample in year 1 and 16.4% in year 4.	To assess the effect of statin on pain, function and radiographic changes in knee joint diagnosed by Kellen-Lawrence (K-L) radiographic grade	Statin use was not associated with knee pain, function or structural progression after a 4-year follow-up period.
Clockaerts et al. (2012)^56^	Prospective population-based cohort study, 2921 participants aged > 55 years old were followed for 6.5 years	To determine association of statin therapy with incidence and progression of knee osteoarthritis (KOA) and hip OA diagnosed by K-L score	In statin users, progression of KOA decreased by adjusted OR = 0.43 (0.25–0.77), but statins use was not associated with overall progression of hip OA
Beattie et al. (2005)^60^	Longitudinal study, 5674 participants of the Study of Osteoporotic Fracture were followed between year 6 and year 8.	To determine the association between using statin and development of hip OA in subjects without baseline disease and progression of radiographic hip OA in elderly women with hip OA based on the number and type of individual radiographic features.	After an average period of 8 years, there was a trend toward decreased progression of radiographic hip OA in statin users. But in subjects without baseline OA statin use was associated with increased risk of new radiographic incident hip OA by OR = 1.95(1.03–4.43).
Sarmanova et al. (2020)^59^	PSM longitudinal cohort study of 178 467 statin users vs. 178 467 non-users	To examine association between statin use and risk of joint replacement surgery due to OA and rheumatoid arthritis.	Statin at high intensity (potency to reduce LDL by 42–55%) reduced the risk of hip or knee replacement surgery only in RA by 23% but not in OA.

a Total hip/knee arthroplasty (THA/TKA)

b PSM = Propensity-score matched

Similarly, a decreased risk of radiographic progression of OA was observed in participants of the Osteoarthritis Initiative study. In this study, statins use decreased the risk of OA progression only in Heberden node-positive patients but not in Heberden node-negative patients.^57^ Inconsistent findings in this study may indicate that the two groups of hand OA differ in regard to mechanisms and the associated factors of progression.

Two studies found decreased risk of revision hip/knee arthroplasty in statin users versus non-users. In these studies, in patients with previous THA/TKA operation, requirement for hip or knee replacement surgery decreased significantly in statins users.^58,59^ Similarly, in Osteoporotic Fracture study there was a trend toward decreased risk of progression of radiographic hip OA in elderly statin user women who had OA at baseline, whereas in subjects without baseline OA, taking statins was associated with 1.95 times (1.03–4.43) increased risk of developing new incident hip OA.^60^

In contrast, in a 3-year longitudinal study comprised 336 participants of the placebo arm of the SEKOIA trial, statin use was associated with a significant radiologic progression of JSN in statin users versus non-users.^61^ Conflicting results of this study have been attributed to several possibility including reverse causality, side effects of statins (muscle pain and weakness). However, the study lacked data regarding severity of hyperlipidaemia, dosage and duration of treatment, cumulative dose of statin. These factors as well as hyperlipidaemia itself can influence on OA progression.^25^

On the other hand, in Osteoarthritis Initiative Study, use of statin over a 4-year follow-up period was not associated with OA progression.^44^ This study also had limitations regarding lack of OA at baseline in a significant proportion of participants and using K-L score for assessment of OA progression, which both conditions are subject to biases and underestimation of the results.

Also, a pooled analysis of 4 population-based large cohorts comprised 132,607 persons found no significant difference in risk of consultation or surgery of KOA or hip OA between non-users and current users of statins for 7.5-year follow-up period.^62^

To summarise, although the study designs varied widely, but most studies found a decreased risk of OA progression in statin users.

## LIMITATIONS

The results on the association between statin therapy and OA vary across various studies. Discrepancies on the risk of development or progression of OA can be attributed to several factors in particular to the study design, patient selection, and variations in methods used for the diagnosis of OA, the type of statins, dosage and duration of treatment and outcome measures. The observational research -either prospective or retrospective- does not benefit from randomization, and therefore the results are prone to selection bias, and so there is a tendency to overestimation or underestimation of the values of population.

All studies in this review compared OA outcomes between statin users and non-users. The case groups who were taking statins for dyslipidaemia were compared with the control group who did not require statins. However, absence of hyperlipidaemia has not been shown in the control groups of previous studies, since hyperlipidaemia in itself is a risk factor of osteoarthritis.^25^ Therefore, the presence of hyperlipidaemia in statin non-user control group can result in OA progression and results in underestimation of treatment response in case group.^51,59^ Variations in clinical response to statins may be also explained by the presence factors other than synovitis which are the source of pain in OA but not responsive to anti-inflammatory effect of statins.^45^ Various outcomes and different measures for assessment of treatment outcomes could be also sources of discrepancies between studies. The sensitivity of measures such as K-L score or JSN which have been used for the evaluation of radiographic progression in these studies can differently affect outcome measure and lead to contradictories. The K-L score is dependent to osteophytes rather than JSN, therefore, it is not sensitive enough to small structural changes, but in contrast JSN is more sensitive than K-L score, hence it has been considered as a gold standard measure for the detection of radiographic changes, and also to show the protective effect of treatments.^61^ Similarly, other outcomes such as risk of consultation for surgery, or surgery of KOA or hip OA, risk of joint replacement surgery or risk of revision of hip/knee arthroplasty following THA/TKA, and the time of consultation for replacement surgery, which have been used to show preventive effect of statins in some studies, are largely dependent to individual decision of treating physician or patient symptoms, and so can differently confound the results of studies and lead to biases.

Recent observations indicate that OA manifests with several phenotypes in terms of clinical features. The presence of inflammatory characteristics such as synovitis, joint effusion, inflammatory pain and stiffness (subgroup with inflammatory phenotype) implicates a therapeutic strategy, but this type of presentation is temporal and does not persist in all stages of the OA process.^63^ Therefore, varieties in distributions of OA phenotypes are also a source of bias, because the aetiology and mechanisms of OA progression and thus response to treatment differ across various phenotypes.

The impacts of factors such as patient selection, retrospective study design, patient adherence to long-term statin therapy, and several unmeasured variables including patient characteristics, severity of OA at baseline, site of OA may be the source of biases and confound the results.

## CONCLUSION

Regarding an important contribution of systemic inflammation in the development and progression of OA, using statins as an anti-inflammatory medication to prevent development or progression of OA is reasonable. In particular, elderly people with OA, both atherosclerosis and metabolic syndrome are prevalent. Taking statins for primary or secondary prevention of atherosclerotic coronary heart disease, can provide additional beneficial effects against OA progression. However, the dosage and duration of treatment with statins as well as the magnitude of efficacy against OA are unclear, and identification of patients who can achieve the most therapeutic benefits is also challenging.

Among several phenotypes of OA, only inflammatory phenotype who present with synovitis are expected to get most benefit from statin therapy. Although the diagnosis of synovitis even at early stage of OA is possible by using sensitive imaging methods such as MRI/US, but in daily clinical practice application of these measures is neither possible nor economical. Thus, identification of clinical synovitis based on the presence of joint pain, stiffness, effusion, and swelling indicates inflammatory phenotype and a possible responsiveness to statin therapy. However, these characteristics which implicates a therapeutic strategy are temporal and do not persist in all stages of OA process.

In general, obese people and diabetics as well as subjects with MetS and cardiovascular disease who have coexistent OA are more eligible to be considered for statin therapy, since these people are more likely to have OA and are also at greater risk of future development of cardiovascular complications. Hence, statin therapy in these populations is widely advised regardless of the presence or absence of OA.

Current data regarding efficacy of statins in OA were provided from retrospective observational studies which are often prone to selection bias, recall bias, and mis-classification bias, and the study populations of these studies do not represent general population. Therefore, this issue requires further studies, especially randomised clinical trials in which OA symptoms and progression being compared between statin user and a placebo group. Nonetheless, unless a formal analysis with weight analysis is made, a conclusion cannot be drawn.
